# Lipid metabolism at the intersection of rheumatoid arthritis and atherosclerosis

**DOI:** 10.3389/fimmu.2026.1790634

**Published:** 2026-04-13

**Authors:** Xiaomei Fang, Huiqi Chen, Xi Yang, Wanying Du, Shushu Wang, Xiaole Yang, Tianfeng Lao, Wensheng Chen, Yingying Shi, Jing Tian, Feng Chen, Guangming Pan, Chunping Liu

**Affiliations:** 1 Department of Critical Care Medicine, Guangzhou University of Chinese Medicine Dongguan Hospital, Dongguan, China; 2 State Key Laboratory of Traditional Chinese Medicine Syndrome, The Second Affiliated Hospital of Guangzhou University of Chinese Medicine, Guangzhou, China; 3 Fifth Clinical Medical College, Guangzhou University of Chinese Medicine, Guangzhou, Guangdong, China; 4 College of Basic Medicine, Guangzhou University of Chinese Medicine, Guangzhou, Guangdong, China; 5 Chinese Medicine Guangdong Laboratory, Guangzhou University of Chinese Medicine, Zhuhai, Guangdong, China; 6 Second Clinical Medical College, Guangzhou University of Chinese Medicine, Guangzhou, Guangdong, China; 7 Department of Cardiovascular Medicine, The Second Affiliated Hospital of Guangzhou University of Chinese Medicine, Guangzhou, Guangdong, China; 8 Shantou Central Hospital, Shantou Clinical Medical College of Jinan University, Shantou, China; 9 Dongguan Hospital, Guangzhou University of Chinese Medicine, Dongguan, China; 10 School of Biology and Food Engineering, Suzhou University of Technology, Changshu, Jiangsu, China; 11 Shenzhen Hospital, Beijing University of Chinese Medicine, Shenzhen, China

**Keywords:** atherosclerosis, cardiovascular risk, comorbidity, inflammation, lipid metabolism, lipid paradox, rheumatoid arthritis

## Abstract

Rheumatoid arthritis (RA) is a systemic autoimmune disease characterized by chronic, symmetric polyarticular synovitis, with a global prevalence of approximately 0.5%-1%. Its pathogenesis involves a complex interplay of genetic and environmental factors, as well as abnormal immune activation. RA patients face a significantly increased risk of cardiovascular disease, with atherosclerosis (AS) and its complications being the leading cause of mortality. Chronic systemic inflammation has long been considered the core pathological bridge linking RA and AS, whereby inflammatory cytokines drive cardiovascular events by impairing endothelial function and promoting arterial plaque formation and destabilization. However, recent research has yielded critical breakthroughs, revealing that dyslipidemia plays a vital role in RA pathogenesis and its comorbidity with AS. It goes beyond a traditional secondary effect, serving as an active participant intertwined with the immune-inflammatory network. This review specifically focuses on lipid-immune crosstalk in RA-AS comorbidity. To this end, we aim to systematically outline the epidemiological evidence for this association, summarize current clinical management strategies and their impact on cardiovascular risk, analyze shared risk factors, and explore in depth the central role of lipid metabolism in their shared pathophysiological mechanisms. We focus on cutting-edge topics such as the “lipid paradox” phenomenon, lipoprotein dysfunction, lipid metabolic dysregulation in macrophages and the imbalance of bioactive lipid mediators to provide a comprehensive perspective and theoretical basis for understanding their common pathophysiological pathways and developing novel therapeutic strategies targeting the metabolism-immune axis.

## 1 Background

Rheumatoid arthritis (RA) is a complex systemic autoimmune disease, with core pathological changes such as abnormal synovial hyperplasia and chronic inflammatory cell infiltration, ultimately leading to progressive cartilage and bone destruction, joint deformity, and functional loss. Its immunopathogenesis involves aberrant activation and interaction of various immune cells. For example, antigen-presenting cells activate T lymphocytes, which secrete copious proinflammatory cytokines such as interferon-γ (IFN-γ) and interleukin 17 (IL-17). Moreover, activated B lymphocytes produce autoantibodies, including rheumatoid factor and anti-citrullinated protein antibodies, that form immune complexes within joints and may be directly pathogenic. Macrophages and fibroblast-like synoviocytes are persistently activated in the RA synovium. They release key proinflammatory mediators including TNF-α, IL-6 and IL-1β. These mediators create a complex inflammatory network that extends systemically beyond the joints (1–3). This systemic inflammatory state closely links RA with various extra-articular manifestations, among which cardiovascular system involvement is the most severe and common, significantly increasing patient disability and mortality (4). Atherosclerosis (AS), a chronic inflammatory vascular disease, shares numerous similar inflammatory pathways and immune cell involvement patterns with RA, laying the foundation for understanding their comorbidity.

In recent decades, chronic inflammation has been viewed as the core driver of premature and accelerated AS in RA patients. Sustained inflammatory cytokine storms induce vascular endothelial dysfunction, and increase permeability (5). They promote the adhesion of circulating leukocytes to the activated endothelium and their subendothelial transmigration. Additionally, they stimulate the proliferation, migration, and extracellular-matrix remodeling of vascular smooth muscle cells. These effects collectively initiate and exacerbate the formation, progression, and destabilization of atherosclerotic plaques. However, the traditional inflammation-driven view cannot fully explain all cardiovascular risk in RA patients. Recently, the research focus has shifted significantly. RA patients commonly exhibit complex and unique patterns of dyslipidemia characterized not only by altered lipid levels but also by profound changes in lipoprotein structure, composition, and function, together with an imbalance of bioactive lipid mediators (6). This lipid disorder is not merely a passive consequence of systemic inflammation; it may actively drive immune regulation and vascular injury, thereby shaping disease progression (7, 8). For example, dysfunctional high-density lipoprotein (HDL) may lose its cardioprotective effects or even transform into a proinflammatory particle, whereas oxidized low-density lipoprotein (oxLDL) can directly activate immune cells and promote foam cell formation (9, 10). Therefore, dyslipidemia is now recognized as another critical hub connecting RA and AS, independent of traditional inflammatory pathways. It forms a complex network of interactions between immunity, metabolism, and vascular function alongside inflammatory signals, opening new avenues for comprehensively understanding RA-related cardiovascular comorbidities and exploring new intervention targets.

## 2 Literature search strategy

Literature searches were conducted in the PubMed database for articles published from 2010 to 2025, using the key terms including “Rheumatoid arthritis”, “Atherosclerosis”, “Lipid metabolism” and “Lipid-immune crosstalk”. Original research articles, meta-analyses and high-quality review articles were included to ensure the scientific rigor and comprehensiveness of the literature basis for this review.

## 3 Epidemiology of the RA-AS association

A clear epidemiological association exists between RA-AS comorbidity, which is supported by substantial evidence from observational and cohort studies, as well as meta-analyses. As early as the late 20th century, pioneering studies suggested a greater risk of myocardial infarction and other cardiovascular events in RA patients than in the general population. Subsequent research confirmed and quantified this risk. For example, a meta-analysis involving over 100,000 patients revealed that the hazard ratio for cardiovascular events in RA patients, adjusted for age and sex, was approximately 1.5-2.0 times greater than that of the general population (11). A 15-year longitudinal prospective cohort study explicitly identified RA as an independent risk factor for cardiovascular disease, with a risk increase comparable to that of type 2 diabetes, highlighting the importance of RA itself as a cardiovascular risk-enhancing factor (12). This association is consistent with a dose-response relationship, wherein patients with higher RA disease activity, greater systemic inflammatory burden, longer disease duration, or additional traditional cardiovascular risk factors exhibit correspondingly faster progression of subclinical AS and a greater risk of major adverse cardiovascular events (MACE) (13). Furthermore, specific autoantibody profiles, especially anti-cyclic citrullinated peptide antibody positivity, are associated with more significant markers of subclinical AS and higher incidence rates of cardiovascular events, suggesting that anti-citrullinated protein antibodies (ACPAs) may not only participate in joint destruction but also act directly or indirectly on the vascular system, exacerbating vascular injury (14, 15).

Despite ample evidence of increased cardiovascular risk in RA patients, the relative weight of traditional versus RA-specific risk factors remains disputed and requires further study. A study revealed that traditional risk factors still contribute significantly to cardiovascular risk in patients, and models that include only traditional risk factors can preliminarily distinguish high-risk populations (16). However, most high-quality systematic reviews and large prospective cohort studies consistently show that RA, especially with persistently active disease, seropositivity or extra-articular manifestations, is independently and significantly associated with an increased risk of major endpoints of atherosclerotic cardiovascular disease, including myocardial infarction, heart failure, ischemic stroke, and cardiovascular death (15, 17, 18). Additionally, the prevalence of lipid metabolism-related comorbidities such as metabolic syndrome, insulin resistance, and nonalcoholic fatty liver disease is significantly greater in RA patients than in healthy individuals, further exacerbating their cardiovascular risk burden and forming a cluster of risk factors centered on inflammation and metabolic dysregulation (19, 20). Recent studies have further revealed the shared inflammatory mechanisms between RA and atherosclerotic cardiovascular disease, and confirmed the differential vascular effects of different TNF and non-TNF biologics on RA patients. TNF inhibitors show a more significant improvement in vascular endothelial function and plaque stabilization compared with non-TNF biologics, which further enriches the epidemiological evidence of RA-AS comorbidity (21).

## 4 The role of lipid metabolism in RA and AS

### 4.1 Lipid metabolism and RA

**Figure 1:**
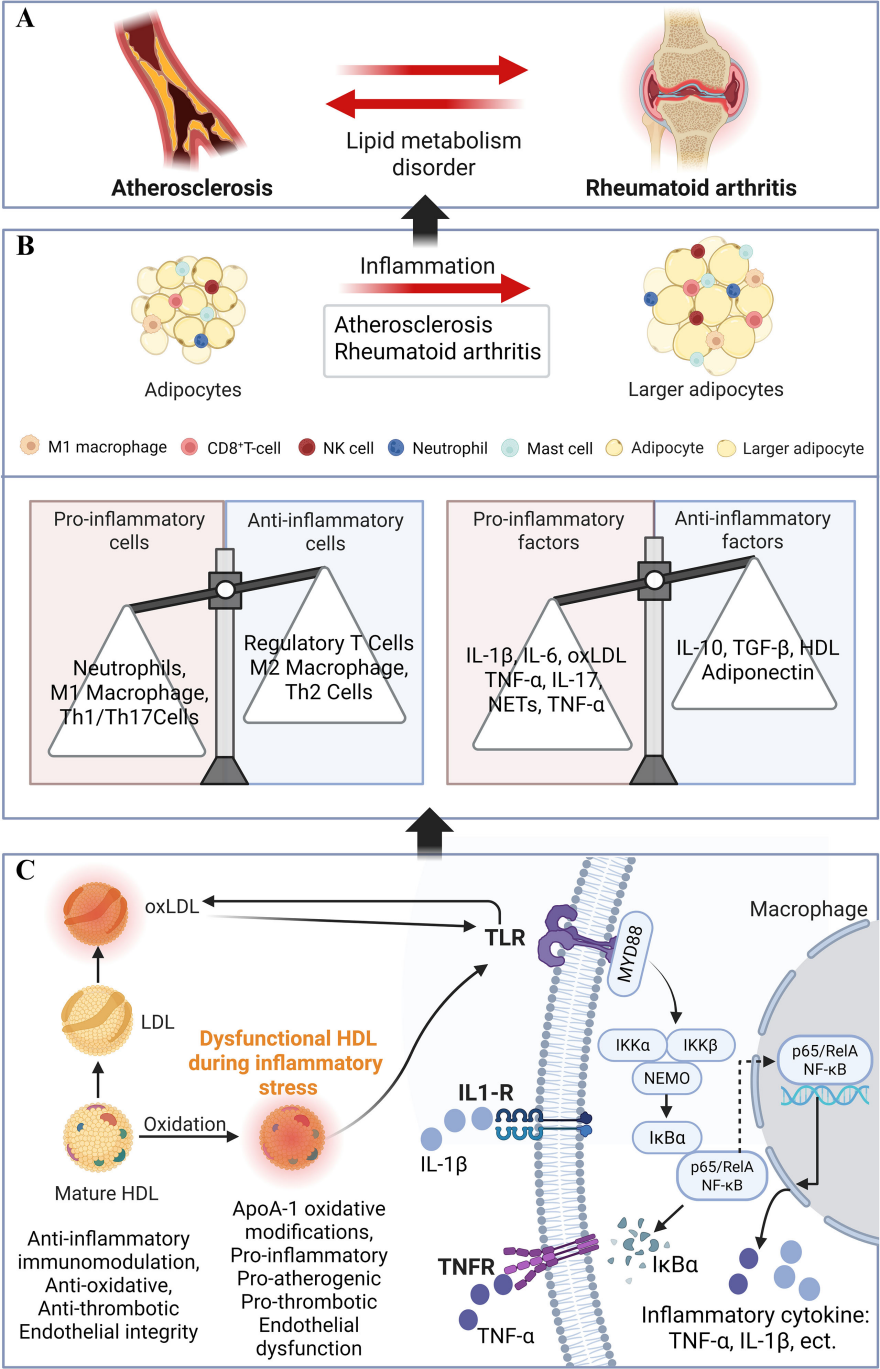
Lipid metabolism-inflammation cross-regulatory mechanism in the comorbidity of rheumatoid arthritis and atherosclerosis. **(A)** Lipid metabolism disorders serve as the core pathological link connecting atherosclerosis and rheumatoid arthritis. A mutually promoting bidirectional association can form between the two diseases and is one of the key links driving the occurrence and development of their comorbidity. **(B)** Normal adipocytes transform into larger adipocytes under inflammatory stimulation, accompanied by a bidirectional imbalance of immune cells and inflammatory factors-proinflammatory cells account for a greater proportion than anti-inflammatory cells; the levels of proinflammatory factors increase, while the levels of anti-inflammatory factors are relatively insufficient. This synergistic effect jointly mediates the comorbidity progression of atherosclerosis and rheumatoid arthritis. **(C)** Mature HDL, which originally exerts protective effects (e.g., anti-inflammatory and immunomodulatory effects), undergoes ApoA-I oxidative modification under inflammatory stress and transforms into dysfunctional HDL, which exhibits pathological properties (e.g., proinflammatory and proatherosclerotic effects); moreover, LDL is oxidized to form oxLDL. oxLDL, IL-1β, and TNF-α bind to TLR, IL-1-R, and TNFR on the macrophage surface, respectively, and initiate the downstream NF-κB signaling pathway via MyD88. The inhibitory protein IκBα is degraded, and the p65/RelA subunit of NF-κB translocates into the nucleus, driving the transcription and release of inflammatory cytokines to further amplify the inflammatory response. oxLDL, Oxidized Low-Density Lipoprotein; LDL, Low-Density Lipoprotein; HDL, High-Density Lipoprotein; TLR, Toll-Like Receptor; MyD88, Myeloid Differentiation Primary Response 88; IL-1-R, Interleukin-1 Receptor; IL-1β, Interleukin-1β; TNFR, Tumor Necrosis Factor Receptor; TNF-α, Tumor Necrosis Factor-α; IKKα, IκB Kinase α; IKKβ, IκB Kinase β; NEMO, NF-κB Essential Modulator; IκBα, Inhibitor of NF-κB α; NF-κB, Nuclear Factor-κB; ApoA-I, apolipoprotein A-I; IL-6, Interleukin-6; IL-17, Interleukin-17; NETs, Neutrophil Extracellular Traps; IL-10, Interleukin-10; TGF-β, Transforming Growth Factor-β.

Compared with healthy individuals, RA patients exhibit significantly accelerated progression of AS. However, traditional lipid indices such as total cholesterol and LDL-C are not significantly elevated in some RA patients and may even be lower than those in healthy individuals (22). This phenomenon, which cannot be fully elucidated by conventional lipid metabolism theories, represents a typical “lipid paradox” in the field of RA. This paradoxical phenomenon profoundly suggests that in the context of RA, relying solely on traditional lipid level measurements is insufficient for accurate cardiovascular risk assessment, and changes in lipoprotein particle quality and function may be more critical. This paradox is increasingly understood as a continuous pathophysiological cascade in which systemic inflammation simultaneously lowers circulating TC and LDL-C through enhanced lipoprotein catabolism, while qualitatively remodeling HDL into a pro-inflammatory, oxidation-prone particle with impaired cholesterol efflux capacity. Normal HDL possesses multiple cardioprotective functions, including anti-inflammatory activity, antioxidant capacity, and the promotion of cholesterol efflux (23). However, in the chronic inflammatory environment of RA, the proteome and lipidome of HDL undergo changes, such as the replacement of apolipoprotein A-I, reduced antioxidant enzyme activity and the incorporation of acute-phase proteins. Studies in RA have shown that inflammatory cytokines such as IL-6 and TNF-α drive loss of apoA-I and antioxidant enzymes like PON1 and enrichment of acute-phase proteins within HDL, yielding so-called pro-inflammatory HDL that no longer protects against LDL oxidation and may actively promote AS by facilitating LDL oxidation, foam cell formation and plaque development (6, 24). These alterations lead to diminished cholesterol efflux capacity and potentially acquisition of proinflammatory properties, which transforms HDL from a vascular protector to a potential disruptor (25) (**Figure 1**). Consistent with this, cohort data indicate that higher inflammatory burden combined with lower HDL-C, rather than higher LDL-C, is associated with an increased incidence of myocardial infarction and cardiovascular events in RA (24, 26, 27).

With respect to LDL, lipoproteins in RA patients are more susceptible to oxidative modification by reactive oxygen species, thereby generating oxLDL. oxLDL is highly proinflammatory and proatherogenic (28) and can be recognized and extensively taken up by scavenger receptors on macrophages. This process is not subject to negative feedback regulation by the intracellular cholesterol content, thereby greatly promoting foam cell formation, which serves as the earliest cellular hallmark of AS plaques (29). Furthermore, the proportion of small and dense LDL subspecies is increased in RA patients (6). These smaller, denser particles penetrate the vascular endothelial barrier more easily and are retained longer in the subendothelial space. Moreover, they are more susceptible to oxidation due to their weaker intrinsic antioxidant capacity, making them more atherogenic than large, buoyant LDL subspecies (30, 31). As key innate immune cells, macrophages play a central role in both RA synovial tissue and AS arterial plaques (32). Within the RA inflammatory microenvironment, the capacity of macrophages to take up modified lipoproteins is increased, whereas the expression of key transporters mediating cholesterol efflux may be decreased due to inhibition by inflammatory cytokines. These perturbations lead to massive intracellular cholesterol accumulation and the eventual transition to a foam cell-like phenotype (10)(**Figure 1**). Studies have confirmed that macrophages derived from the peripheral blood monocytes of RA patients exhibit impaired cholesterol efflux capacity, and this functional defect is positively correlated with clinical disease activity, providing a mechanistic explanation for accelerated AS in RA patients (33). Beyond macrophages, oxLDL and other post-translationally modified LDL particles in RA also interact with endothelial cells, T cells and vascular smooth muscle cells (VSMCs) in a highly cell type-specific manner. On endothelial cells, oxLDL binding to LOX-1 activates ROS-dependent NF-κB signaling, decreases eNOS-derived NO, and upregulates adhesion molecules like VCAM-1 and ICAM-1, and colony-stimulating factors, thereby promoting monocyte adhesion and trans-endothelial migration (34). In T cells, oxLDL and oxidized phospholipid epitopes are taken up and presented by dendritic cells, driving Th1/Th17-skewed responses; oxidized and electronegative LDL subfractions further enhance expression of activation markers and tissue factor on T cells, amplifying vascular inflammation and thrombogenicity (35). In VSMCs, modified LDL and their lipid products are internalized via scavenger receptors such as SR-A and LOX-1, inducing phenotypic switching toward a synthetic, foam-cell-like state with increased extracellular matrix and tissue factor production, which contributes to necrotic core expansion and fibrous cap weakening (36). Thus, RA-associated modified LDL species interact with distinct receptor-signaling modules in macrophages, T cells, endothelial cells and VSMCs, thereby linking joint inflammation to the accelerated development of AS.

In addition to classical lipoprotein metabolism, research into the network of bioactive lipid mediators has provided new perspectives for understanding immune-metabolic crosstalk in RA. In fact, lipids not only serve as energy stores or membrane structural components but also generate metabolites that act as crucial signaling molecules, finely modulating the initiation, amplification, and resolution of inflammation (37). N-6 polyunsaturated fatty acids (n-6 PUFAs), particularly C20 subtypes such as arachidonic acid (20:4n-6), are highly enriched in RA synovial tissue and fluid. The derived proinflammatory lipid mediators, including prostaglandin E2 (PGE2) and leukotriene B4 (LTB4), can powerfully drive local and systemic inflammation by recruiting and activating immune cells such as neutrophils and macrophages (38). Conversely, pro-resolving lipid mediators derived from omega-3 polyunsaturated fatty acids play a key role in actively terminating inflammation and promoting tissue repair and functional recovery. Studies have confirmed that the levels of these pro-resolving mediators are significantly lower in RA patients than in healthy individuals, leading to impaired inflammation-resolving programs and perpetuation of chronic joint inflammation (39, 40). The imbalance between proinflammatory and pro-resolving lipid mediators forms a molecular bridge connecting lipid metabolism to chronic inflammatory diseases and offers potential new targets for intervening in the lipid mediator network via nutritional or pharmacological means to treat RA and its cardiovascular comorbidity.

### 4.2 Lipid metabolism in the pathogenesis of AS

Dyslipidemia remains central to the pathogenesis of AS. Recent research has vastly expanded our understanding of the roles of various lipoproteins and their subfractions in AS, thus moving beyond the oversimplified binary dichotomy that categorizes LDL-C as “bad cholesterol” and HDL-C as “good cholesterol.” Among these, triglyceride-rich lipoproteins (TRLs) and non-HDL cholesterol (non-HDL-C) have been established as potent, independent AS risk factors (41) (**Figure 1**). Even when a patient’s LDL-C is maintained at target concentrations with statin therapy, elevated levels of remnant cholesterol can still facilitate significant cholesterol deposition in the arterial wall. These particles exhibit proinflammatory characteristics, which activate endothelial cells and macrophages to sustain the progression and instability of atherosclerotic plaques (42). In addition, a subset of RA patients presents with a lipid profile characterized by high triglycerides and low HDL-C, which reflects elevated TRLs and remnant cholesterol. These patients display higher concentrations of TNF-α and chemokines, alongside reduced paraoxonase-1 activity and antioxidant capacity. Furthermore, they exhibit a poorer clinical response to TNF-α blockade, reinforcing that TRLs and remnant particles are inflammation-sensitive, proatherogenic lipoproteins in RA. Lipoprotein(a) [Lp(a)] is a unique lipoprotein whose levels are largely genetically determined, vary greatly between individuals, and are minimally affected by conventional lifestyle interventions or most lipid-lowering drugs (43). Elevated Lp(a) is an independent risk factor for AS, coronary artery disease, aortic valve calcification, and ischemic stroke (44, 45). Research indicates that Lp(a) levels may change under inflammatory conditions, and its role in cardiovascular risk stratification and prediction in RA patients is gaining increasing attention (46).

The conceptualization of HDL-C as a therapeutic target has undergone a paradigm shift in clinical practice from a quantitative increase to a functional improvement. Although epidemiological surveys consistently show an inverse correlation between HDL-C levels and cardiovascular risk, several large clinical trials aiming to substantially increase HDL-C pharmacologically have failed to demonstrate significant cardiovascular endpoint benefits (47, 48). This outcome shifted the research focus from increasing HDL quantity to improving HDL functional quality, such as enhancing cholesterol efflux efficiency, anti-inflammatory capacity, and antioxidant activity. Concurrently, a series of new lipid-lowering therapeutic targets have emerged. Angiopoietin-like protein 3 (ANGPTL3), which is expressed primarily in the liver, inhibits lipoprotein lipase and endothelial lipase activity. Monoclonal antibodies or small interfering RNAs targeting ANGPTL3 have been shown to effectively reduce LDL-C, HDL-C, and triglyceride levels in patients with homozygous familial hypercholesterolemia, suggesting new options for patients with refractory dyslipidemia (49, 50). Furthermore, high-dose prescription-grade icosapent ethyl (an ethyl ester of eicosapentaenoic acid, EPA) demonstrated in large randomized controlled trials such as REDUCE-IT that it could further reduce the risk of MACEs in patients with hypertriglyceridemia on statin therapy. Its mechanism is thought to not rely solely on triglyceride-lowering effects but may involve multiple beneficial effects, including anti-inflammatory, antioxidant, improved endothelial function, and plaque stabilization effects (51). These advances not only confirm the feasibility of targeting other lipid-related pathways but also highlight the central role and broad prospects of lipid metabolism management in AS prevention and treatment.

## 5 Assessment of comorbidity risk factors

The risk of AS in RA patients is driven by a complex network comprising traditional cardiovascular risk factors and RA-specific disease factors. Smoking is one of the most prominent traditional cardiovascular risk factors (52). It is not only a key environmental trigger for RA onset, which may initiate autoimmunity via mechanisms such as protein citrullination but also a potent promoter of AS (53, 54). Smoking induces systemic oxidative stress and endothelial dysfunction, exacerbates the inflammatory state, and may synergize with RA-specific antibodies to further increase the risk of vascular damage. Obesity and metabolic syndrome are significantly more prevalent in RA patients than in the general population, indicating substantial pathophysiological significance (55). Adipose tissue, especially visceral fat, is a highly active endocrine and immune organ that secretes various bioactive adipokines, such as leptin and proinflammatory cytokines, into the circulation, thereby exacerbating chronic low-grade inflammation and insulin resistance and providing a pathological basis for AS development (56, 57). Furthermore, the comorbidity rates of hypertension and diabetes are higher in RA patients than in the age-matched general population. These disease states interact with the inherent inflammation and immune dysregulation of RA, creating a vicious cycle that accelerates vascular pathology (58).

Among RA-specific risk factors, disease activity and duration are the most powerful predictors (59). Autoantibodies are associated with more erosive joint disease and poorer joint outcomes. Increasing evidence indicates that ACPA-positive status, especially at high titers, is associated with increased arterial stiffness, elevated levels of inflammation and endothelial activation markers, as well as an increased risk of subclinical atherosclerosis (60, 61). This strongly indicates that ACPAs may actively participate in vascular injury processes, possibly by recognizing citrullinated proteins in the vascular wall or through other direct mechanisms (14). In addition to ACPA, several studies have shown that the positive and high titer status of rheumatoid factor (RF) is also related to subclinical atherosclerosis markers such as thickening of carotid intima-media and plaque formation, suggesting that seropositive RA patients bear a higher burden of AS. Especially when high titers of RF and ACPA are present simultaneously, AS related genotypes such as OPG and higher carotid intima-media thickness are clustered. In patients with active RA, the anti-inflammatory benefits of low-dose glucocorticoids used to achieve rapid disease control may outweigh their short-term metabolic adverse effects without conferring significant short-term cardiovascular risk (62, 63). Therefore, clinical practice requires careful benefit-risk assessment tailored to the individual patient, aiming for the lowest effective dose and shortest possible duration (64). Identifying and actively managing these modifiable risk factors is crucial for reducing the incidence of cardiovascular complications and improving the long-term prognosis of RA patients. For example, smoking cessation can be promoted through education and medication, and weight control and blood pressure control can be achieved through dietary and exercise interventions. Moreover, a personalized approach is required for anti-RA treatment to achieve and maintain low disease activity or clinical remission (65, 66).

## 6 Treatment strategies for RA-AS comorbidity

Given the markedly elevated cardiovascular risk in RA, major international rheumatology and cardiology guidelines designate RA as an important risk-enhancing factor for atherosclerotic disease (67, 68). Consequently, routine cardiovascular risk assessment is advised for every RA patient, with systematic monitoring of blood pressure, fasting glucose, and lipid profiles and ultrasound-based screening for subclinical AS, such as carotid intima-media thickness (69).

In terms of treatment strategies, effective and sustained control of RA disease activity is the cornerstone for reducing cardiovascular risk. However, the impact of different classes of antirheumatic drugs on cardiovascular outcomes is heterogeneous, making them crucial considerations in clinical decision-making (70). The mechanisms, clinical benefits and supporting evidence of different classes of drugs for RA-AS comorbidity are systematically summarized in **Table 1**. Methotrexate, the anchor drug in RA treatment, has been shown by large-scale, long-term real-world studies and meta-analyses to be associated with reduced risks of myocardial infarction and all-cause mortality, primarily because of its potent systemic anti-inflammatory effects (71, 72). However, methotrexate may increase homocysteine levels, theoretically predisposing patients to endothelial injury, and monitoring for hepatotoxicity and myelosuppression during prolonged use is needed (73, 74).

**Table 1:** Medications for RA-AS comorbidities.

Drug classification	Drug	Mechanism	Benefit	Evidence type	Ref
Small molecular compounds	Methotrexate	Delivers potent systemic anti-inflammatory effects and inhibits the abnormal activation of immune cells.	Among patients with RA receiving biologics, concomitant methotrexate use may be associated with lower risk for CVD events.	Cohort study	(83)
	Sulfasalazine	Inhibits the production of pro-inflammatory cytokines, suppresses vascular smooth muscle cell proliferation and controls neointimal hyperplasia.	The sulfasalazine-containing triple csDMARD strategy could reduce 18F-FDG uptake in the carotid/aorta over 6 months, indicating its beneficial role in RA with latent AS.	Randomized controlled trial	(84, 85)
	Leflunomide	Inhibits dihydroorotate dehydrogenase, suppresses lymphocyte proliferation and inflammatory responses, and modulates inflammatory pathways that are related to lipid metabolism.	In the RA population, continued use of leflunomide may be associated with an increased risk of acute myocardial infarction, alerting to the need to be aware of potential CVD risk.	Nested case-control study	(86, 87)
	Hydroxychloroquine	Inhibits lysosomal function to reduce immune cell activation and has antioxidant effects that protect vascular endothelial cells.	In RA patients, the use of hydroxychloroquine is associated with a reduced risk of cerebral infarction and overall MACE.	Cohort study	(88–91)
Biologic agents	Etanercept	Suppresses systemic inflammation, directly improves vascular endothelial function, reduces vascular wall inflammation, and promotes plaque stabilization.	Etanercept, Adalimumab and Infliximab may reduce cardiovascular risk in RA patients, manifested by slowed progression of carotid intima-media thickness and decreased homocysteine levels.	Observational study	(92–94)
	Infliximab				
	Adalimumab				
	Golimumab		In the new-onset RA cohort, use of Golimumab was associated with a relative reduction in risk of MACE of approximately 90%, suggesting a possible protective effect on RA-AS-related outcomes.	Case-control study	(95)
	Certolizumab		Compared to etanercept, RA patients receiving treatment with Cetolizumab did not show an increased risk of ACS.	Cohort study	(96, 97)
	Tocilizumab	Inhibits the IL-6 receptor to exert potent anti-inflammatory effects, enhances reverse cholesterol transport, and modulates the lipid profile.	RA active patients who received Tocilizumab did not have elevated global cardiovascular risk scores and no significant progression in carotid structural parameters at 265 weeks of follow-up.	Cohort study	(98, 99)
	Abatacept	Competitive binding of CD80/CD86, blocking its interaction with CD28, thereby inhibiting the excessive activation of T cells and reducing the release of inflammatory factors.	Compared with traditional csDMARD, the use of abatacept may slow down the progression of carotid plaque scores in RA patients.	Observational study	(100, 101)
	Rituximab	Depletes CD20^+^ and CD19^+^ B cells to reduce autoantibody IgG production and inhibit the autoimmune response in RA.	At a 1-year follow-up, RA patients treated with rituximab showed a significant improvement in HDL-C efflux capacity.	Observational study	(102, 103)
	Sarilumab	Acts as an anti-IL-6 receptor monoclonal antibody to potently inhibit IL-6 signaling and modulates the lipid profile with a mild increase in TC and LDL-C levels.	Sarilumab can additionally reduce HbA1c in RA, especially in patients with diabetes, which may have potential advantages in improving RA and cardiovascular risk factors at the same time.	Randomized controlled trials	(104–106)
tsDMARDs	Tofacitinib	Inhibits the Janus Kinase (JAK) pathway and suppresses abnormal immune activation and inflammatory responses.	Tofacitinib has a balanced effect on lipid profile and some pro AS factors, but did not prevent the progression of carotid intima-media thickness during 1-year follow-up.	Cohort study	(107, 108)
	Baricitinib	Inhibits JAK1 and JAK2 to suppress pro-inflammatory cytokine signaling and improves inflammation-related dyslipidemia.	The incidence of MACE and VTE with Baricitinib remained stable and within the known range for RA patients, indicating a neutral overall risk of cardiovascular events while controlling arthritis.	Randomized controlled trials	(109–111)
	Upadacitinib	Inhibits JAK to block the phosphorylation of downstream effector proteins, also inhibits IL-6-induced inflammatory and angiogenic factors.	The MACE and VTE event rates of Upadacitinib in RA patients are similar to those of Adalimumab, and do not increase with prolonged exposure.	Randomized controlled trials	(112–114)
	Filgotinib	Acts as a selective JAK1 inhibitor to suppress proinflammatory cytokine signaling and improves inflammation-related dyslipidemia.	After years of exposure, Flgotinib has overall good safety and a low incidence of MACE in RA patients.	Randomized controlled trials	(115–117)

tsDMARDs, targeted synthetic Disease-modifying Antirheumatic Drugs; RA, Rheumatoid Arthritis; MACE, Major Adverse Cardiovascular Event; AS, Atherosclerosis; IL-6, Interleukin 6; JAK, Janus Kinase; TC, Total Cholesterol; LDL-C, Low-Density Lipoprotein Cholesterol; CVD, Cardiovascular Disease; csDMARD, conventional synthetic Disease-modifying Antirheumatic Drugs; 18F-FDG, 18F-fluorodeoxyglucose; ACS, Acute Coronary Syndrome; CD20^+^, Cluster of Differentiation 20^+^; CD19^+^, Cluster of Differentiation 19^+^; IgG, Immunoglobulin G; HDL-C, High density lipoprotein cholesterol; VTE, Venous Thromboembolism.

Biological agents have revolutionized RA treatment, and their differential vascular effects on RA patients have been clarified by recent studies, which provides a new basis for selecting biological agents for RA patients with high cardiovascular risk (21), their potential impact on cardiovascular risk has become a research hotspot in recent years. While TNF-α inhibitors effectively control arthritis signs and symptoms, they have been shown in multiple large observational studies and meta-analyses to reduce the incidence of MACEs in RA patients (70). The mechanism may extend beyond suppressing systemic inflammation to include directly improving vascular endothelial function, reducing vascular wall inflammation, and promoting plaque stabilization (75). In contrast, IL-6 pathway inhibitors such as tocilizumab, despite potently suppressing inflammation and significantly improving clinical symptoms, have been used in key randomized controlled trials and long-term follow-up to uniquely affect the lipid profile, causing concurrent increases in total cholesterol, LDL-C and HDL-C (76). Complementary cohort and meta-analytic data in RA further indicate that, despite these lipid changes, IL-6 inhibitors do not confer a higher short- to mid-term risk of major adverse cardiovascular events compared with TNF inhibitors and may even be associated with a lower risk of myocardial infarction in pooled analyses (77, 78). Nevertheless, the extent to which this dissociation between lipid profile modification and vascular biomarkers translates into durable reductions in AS-burden and hard cardiovascular endpoints in RA remains uncertain and will require longer-term, adequately powered outcome studies.

Janus kinase (JAK) inhibitors represent another major advance in RA treatment. The results of large postmarketing safety studies, such as the ORAL Surveillance study, have raised significant concerns. This study revealed that in RA patients with at least one cardiovascular risk factor, the use of certain JAK inhibitors, such as tofacitinib, was associated with a greater risk of MACE and malignancy than the use of TNF-α inhibitors (79, 80). This finding prompted regulatory agencies such as the United States Food and Drug Administration and the European Medicines Agency (EMA) to update prescribing guidelines, emphasizing thorough assessment of cardiovascular and oncologic risks before prescription and prioritizing other treatment options for high-risk individuals (81). But the safety signals of JAK inhibitors are molecule-specific and population-specific, and the cardiovascular risk cannot be generalized to the entire class of drugs. Therefore, RA patients with AS or high cardiovascular risk demand highly individualized treatment strategies from rheumatologists. Moreover, careful weighing of the anti-inflammatory efficacy, potential metabolic impacts, and long-term cardiovascular safety data of different drugs is necessary. Strengthening multidisciplinary collaboration for comprehensive, whole-process patient management is fundamental to achieving optimal long-term quality of life and survival rates (82).

## 7 Summary

RA-AS comorbidity is a complex outcome of multilevel, multifactorial interactions, forming an intricate pathological network in which chronic immune inflammation and complex dyslipidemia are interwoven, mutually reinforcing, and causal. Systemic inflammation is traditionally regarded as the core bridge linking RA to AS. Currently, we increasingly recognize that dyslipidemia not only is a traditional risk factor for AS but also acts as an active regulator within the systemic immune-inflammatory response of RA, and its influence permeates the entire disease course. The “lipid paradox” highlights the importance of assessing lipoprotein function and quality in the context of RA, a finding whose clinical importance far exceeds simple lipid level measurements. These aberrant lipid metabolic pathways collectively constitute drivers that accelerate vascular injury, from dysfunctional HDL to proinflammatory oxLDL and sdLDL, as well as from disrupted cholesterol homeostasis in macrophages to an imbalance between proinflammatory and pro-resolving lipid mediator networks. This work highlights lipid-immune crosstalk as a core pathological hub. Key conceptual advances include summarizing lipid metabolism’s active regulation of immunity, integrating cell-specific lipid-immune mechanisms, and emphasizing metabolism-immune axis targeting potential, all of which underscore how aberrant lipid metabolic pathways collectively drive vascular injury in RA-AS.

In summary, current evidence highlights a complex bidirectional crosstalk between immune activation and disordered lipid metabolism as a central driver of accelerated AS in RA. To translate these insights into clinical benefit, future work should prioritize mechanistically anchored, testable questions. Firstly, to verify the molecular and cellular-level dialog and mutual regulatory mechanisms between different immune cell types and lipid metabolism under specific RA conditions, such as focusing on T cell-macrophage crosstalk and its regulation of cholesterol efflux in RA synovial tissue. Secondly, to screen and validate sensitive, specific biomarkers that reflect lipoprotein function, remnant cholesterol burden, or specific pro-resolving lipid mediator levels in RA patients. Circulating microRNAs or lipid-derived mediators that correlate with disease activity and cardiovascular risk are potential research subjects. Thirdly, to develop and test the efficacy of combination therapeutic regimens like small molecules, biologics and nutritional interventions. For instance, well-designed randomized controlled trials are needed to clarify the long-term impact of different antirheumatic drugs on cardiovascular endpoints in RA patients. The ultimate goal is to maximally improve patients’ long-term quality of life, functional status, and survival rates, embracing the era of precision medicine with its challenges and opportunities.
